# Development of a novel hand cleansing product for low-income contexts: The case of tab soap

**DOI:** 10.1371/journal.pone.0283741

**Published:** 2023-05-31

**Authors:** Edward Brial, Robert Aunger, Winnie Costancia Muangi, Weston Baxter

**Affiliations:** 1 Dyson School of Design Engineering, Imperial College London, London, United Kingdom; 2 Environmental Health Group, London School of Hygiene and Tropical Medicine, London, United Kingdom; 3 School of Economics, University of Dar es Salaam, Dar es Salaam, Tanzania; 4 Department of Economics, University of Reading, Reading, United Kingdom; Cardiff University, UNITED KINGDOM

## Abstract

Handwashing with soap is a widely advocated public health measure, but seldom practiced, partly because it is often difficult (especially outside of rich Western country contexts) to make both soap and water readily available in relevant situations. This study used both Behaviour Centred Design and Human Centred Design to guide development of a novel hand cleansing technology appropriate for the context of post-toilet hand cleansing in resource-poor societies. Extensive prototyping and field testing resulted in the pilot production of ‘tab’ soap, a small but durable single-use, decomposable substrate embedded with soap. It can be produced in dispenser roll or tear-off formats. With this affordable solution, one may use soap without worrying about contamination pretty much anytime and anywhere. A small-scale field test showed that all poor households in rural and peri-urban areas in Tanzania included in the proof-of-concept study (N = 12 households) would use the product reliably over the medium term. Tab soap awaits full-scale production and marketing but could make hand cleansing a more popular practice around the world.

## Background

Handwashing with soap is a highly effective means of reducing the transmission of many infectious diseases [1–3]. However, it is not often practiced, especially in areas where it could do the most good [4]. A major problem is that soap and water are often difficult to manage in situations where households have very limited incomes [5, 6]. Many handwashing systems (facilities, products, or services) have been suggested to reduce the costs and/or increase the value of handwashing, such as ‘tippy-taps’ (modified jerrycan) [7, 8], and Oxfam buckets (a plastic bucket with tap; https://oxfamapps.org.uk/bucket/). However, few studies suggest that these innovations have created lasting effects on consistent handwashing behaviour. Recently developed handwashing facilities have highlighted the importance of working with design professionals and employing several rounds of iterative prototyping and testing to develop functional and desirable options for handwashing. Examples include the ‘Povu Poa’ (‘Cool Foam’) handwashing station in Kenya (http://demandasme.org/cool-foam/), and the ‘Happy Tap’ project in Vietnam: (http://www.wsp.org/sites/wsp.org/files/publications/WSP_Designing_Handwashing_Station_HWWS.pdf). However, they have not proven themselves sustainable at scale (e.g., through development of a profitable business or subsidy extended to a large population over time). Handwashing with soap has long been known as a highly effective means of preventing diarrhoea, especially among the young [9]. The recent COVID-19 global pandemic has further highlighted the importance of handwashing as a public health measure, and the continuing need for improved technologies to deliver this behaviour [10]. This paper describes a case study of a design process to produce a handwashing technology that is grounded in behavioural insight and that is found to be economically viable, technically feasible, desirable, and scalable.

### Tanzanian context

We chose to develop and test a handwashing system in a challenging context: rural and peri-urban Tanzania. This context is challenging because it presents few ecological, economic or technological resources and represents a lack of behavioural compliance despite widespread awareness of the need for handwashing with soap [11, 12]. A recent on-ground study of a typical small town found that only 13% of households had a handwash facility available [13]. Nationally, in 2016, only 7% of households had a fixed place to handwash, with soap and water present [14].

This study was designed to create and test a desirable, feasible and viable post-defecation handwashing system (product and/or service) for households without on-site water connections in low-income contexts like those in parts of Tanzania. Its objectives were to:

1. Realise our aim through a systematic and documented design process following principles of Human Centred Design and Behaviour Centred Design.
2. Develop this handwashing system to the level of a functioning prototype with a clear business case for its viability
3. Assess the effectiveness of the prototype to increase handwashing behaviour over a period of time as a proof of concept.

This research project was nested within a wider project to design and deliver a national sanitation and hygiene behaviour change campaign. This project is led by the Tanzanian Ministry of Health, Community Development, Gender, Elderly, and Children, and supported by the CLEAR consortium–a group of international and Tanzanian experts in behaviour change, sanitation, hygiene, marketing, capacity strengthening, research, and management. Consortium partners include the London School of Hygiene and Tropical Medicine, Innovex Development Consulting, McCann Global Health, EXP Marketing, and Clouds Media Group. The consortium is funded by the UK Foreign, Commonwealth and Development Office (FCDO).

### Theoretical background

To maximize the possibility of success, this study used a novel design approach which combines Behaviour Centred Design (BCD) theory and process with Human Centred Design (HCD) expertise.

#### Behaviour centred design

Behaviour Centred Design (BCD) was developed by academics at the London School of Hygiene and Tropical Medicine and provides a systematic, theoretically driven, qualitative research framework with a five-step design process to **A**ssess, **B**uild, **C**reate, **D**eliver and **E**valuate a behaviour change program or intervention [15]. *Assess* is undertaken to understand what is already known about the problem at hand from experts and the published literature. *Build* augments this knowledge base through further investigation, typically in the field, through formative and other research. *Create* is the step during which the intervention ideas and materials are actually produced. These plans and materials are then *Delivered* in the form of activities or contexts within which the target audience comes into contact with them, hopefully to learn something that changes their expectations as to the value of performing the target behaviour. *Evaluation* is a step in which the program team determines how well the intervention was delivered and what effect it had on desired outcomes, such as use of a new product. Note that the BCD approach suggests you can add a systematic process to creativity, preferably using creative professionals. Each step can be thought of as going through a process of elaboration of creative options and then selection of a particular option to take forward into the next phase, with the most potential to be powerful and effective, along the lines of the ‘double diamond’ approach [16] that sees two rounds of divergent and convergent design activities regarding discovery research, challenge definition, concept development and concept delivery. BCD also provides unique theoretical tools, such as a suggested list of evolved human motives [17] and the concept of a behaviour setting, derived from 1950s ecological psychology [18], which details the various kinds of environmental and psychological factors that impinge to determine behaviour within a circumscribed time and place.

#### Human centred design

Human Centred Design (HCD) is a creative approach to product and service design which emphasizes the human perspective at each stage of the process [19, 20]. The intent of this approach is to enable a better fit between human needs and designed offerings to realise more successful solutions. It contrasts with more traditional technology-centric approaches by emphasizing the context of use over functional features of the product or service. The terminology used by different firms varies but always involves working closely with end users and stakeholders to understand the needs and wants of those who will actually use the offering and their unique contextual needs. This process is commonly broken down into three phases: 1) inspiration which consists of research and synthesis; 2) ideation in which ideas are developed, prototyped and refined; and 3) implementation where the offering is deployed. Each of these phases are iterative in nature and aim to produce an overall creative output.

There are similarities between the HCD approach and the qualitative research methods often used in rigorous behaviour change research (e.g., including the BCD approach). However, HCD tends to be less scientifically protocol-driven, without explicit use of behavioural theories, and with a focus on the design team gaining inspiration or insight from participants, rather than systematic collection and analysis of data to explain or describe behavioural practices in a population. Used together, each approach would seem well suited to filling gaps in the other.

Though both processes were followed throughout the research, the terminology used in this reporting aligns with BCD (i.e., ABCDE steps) for simplicity. The A & B steps of BCD roughly correspond to the Inspiration phase of HCD, the C step of BCD to the Ideation phase of HCD and the D & E steps of BCD to the Implementation phase of HCD. The remainder of this paper will adopt this alignment of the steps and phases in the reporting.

## Methods

This section will describe the methods used to select households to serve in the study, the means developed to test existing technologies, and to develop and test new possible solutions. Development of the novel technology took place across phases of work corresponding to the ABCDE phases of BCD. Each phase involves a different kind of investigation and the methods used to make these investigations. The methods used are dependent on the type of solutions that result from that phase of the design process. The outcomes from these activities will be described in the subsequent sections.

Ethical permission to conduct the field research was obtained from the LSHTM Ethics Committee (reference 15451) and the Tanzanian Ministry of Health (NIMR/HQ/R.8a/VOL. 1X/3122). All senior project members received training on human subject research ethics. All study participants were given a printed information sheet about the study and provided written consent. Where participants could not read, the information sheet was read to them and they were asked if they had any questions before commencing. A thumb print was recorded in place of a signature if they could not write. In a few instances, minors were present with their parents when conducting research. In those cases, consent was collected from their parents on behalf of their children.

### Assess phase

The overarching objective of this phase is to develop a rich initial contextual understanding of the target behaviour. This understanding was achieved through a review of literature and competitive products as well as an inception workshop with relevant stakeholders to further scope the study. Stakeholders included experts from the Centre for Behavioral Studies (CBS) at the University of Dar es Salaam, Muhimbili University of Health and Allied Sciences (MUHAS), Unleashed Africa, Bremen Overseas Research and Development Association (BORDA), Centre for Community Initiatives (CCI), LIXIL, and officials from the Tanzanian Ministry of Health. The result of this phase is preparation for qualitative fieldwork in the Build phase.

### Build phase

Following the Assess Phase, in-depth formative research and testing of existing offerings was conducted in five communities/villages–two rural and three peri-urban–around Dar es Salaam, Morogoro and Mwanza regions, Tanzania. In all locations, a small number of households were selected to participate in the study (less than five in each setting). Settings and households were purposely selected, based on factors such as access to water and income. Eligibility criteria included:

1. No private water connection or piped water on-site (i.e., water for handwashing comes from stored water in the household collected from a shared water source outside of the household, meaning residents either collect the water themselves or pay someone to bring it to them–the majority condition in low-income settings)—to ensure even the poorest households would be represented (40% of urban and 93% of rural households lack access to piped on-site water: https://www.globalwaters.org/resources/assets/tanzania-institutional-framework-water-supply)
2. At least one child under 5 years and at least one child over 5 years in the household (to maximize demographic relevance).

Suitable households were identified through local leaders and guides. Participants were also selected to reflect diversity in social and economic status and hygiene practices. We thus sought to include individuals with unique needs or viewpoints in regard to handwashing in our sample (e.g., elderly, disabled, existing soap users, people with varying occupations, such as teacher, nurse or village chiefs).

A total of fifteen households were included in this phase of research. Five goods sellers and a focus group with local leaders were also interviewed (see Table 1; note use of reference codes when quoting particular findings from formative research). Household visits lasted between 1.5 and 2.5 hours and involved discussions about daily routine, handwashing, water usage, feedback on existing products and responses to potential early product ideas. The main participant was always the woman of the household though men, children or visitors were included as appropriate. Additional research was carried out with sellers of buckets (2) and soap (4) to understand local supply chains and costs within business exchange. Local leaders (5) were interviewed to understand both the perception and influential role they had in the community.

**Table 1:** Build phase study samples.

Location (region)	Reference code	Context	Number of households / participants
Dar es Salaam Households	DSR	Peri Urban	4
Morogoro Households	MG	Rural	4
Dar es Salaam Households	DSS	Slum	3
Dar es Salaam Sellers	DSSS	Slum	1
Mwanza Households	MWS	Slum	2
Mwanza Households	MWR	Rural	2
Mwanza Sellers	MWCS	City	5
Mwanza focus group	MWFG	City	5

The field research team consisted of three researchers from Imperial College London and London School of Hygiene and Tropical Medicine, and two Tanzanian translators and research collaborators with experience in design and water/sanitation/hygiene (WASH) projects. The objectives of this field research were the following:

1. Map behaviour settings and confirm main motives for handwashing in households with no on-site water connections throughout Tanzania.
  - Focus given to props and infrastructure used for handwashing with soap the brief specified the design of a product system.
  - Understanding motivations involved in current handwashing practice and motivations that could be leveraged for novel interventions.
  - Finding behavioural insights. Followed by validation and implementation of insights into building a Theory of Change and design brief to develop concepts.
2. Understand behaviour settings around acquiring water for the household.

The qualitative research (interviews and observations) included methods used in similar research studies by the BCD research team such as Daily Scripting and Behaviour Trials. These methods were further complemented by HCD tools and methods including Technology probes, Persona development and Immersion Exercises. Table 2 provides a summary of each method used and the methodology to which it tends to belong. All methods were used with verbal and written approval.

**Table 2:** Tools/Protocols used during in-depth qualitative research.

Tool/ Protocol	Description	Tool Source	Number of Households
Contextual inquiry	Structured technique of interviewing user’s performing a task in context	HCD	15
Site facilities inventory	Tour of the house to ascertain presence of relevant objects and behaviours.	BCD/ HCD	15
Daily Script	Individuals outline the sequence of activities during a regular day (with emphasis on hygiene practices).	BCD	4
Analogous inspiration	Elicit reactions to products that could inform or inspire the design of the intervention.	HCD	9
Behaviour Trial / User enactment / Technology probes	Develop an understanding of the merits of an intervention through participant role-playing handwashing with an existing technology.	BCD/ HCD	9
Point of Sale	Find soap sellers and ask them about their business model and customer base	HCD	5
Empathy Exercises	Use of the toilet and handwashing facility to better understand how people may think, feel and act in context.	HCD	15
Product Ranking	Participants rank a set of existing products according to various parameters to elicit the perceived merits of these technologies.	BCD/HCD	3

### Create phase

The overall objective of the Create phase is to translate the learnings from the first two phases into a behavioural intervention. The intention was to develop many (100+) ideas and narrow this possible set of solutions to at least three ideas to the point of a fully defined design concept before selecting a final one to take forward for delivery and evaluation. That final concept was prototoyped and refined through feedback in the field before further development through additional in-field testing. The rationale for this was to increase creative input and mitigate risk of any one idea not working in context.

The Create activities were structured to generate and refine a set of creative interventions with both theoretical and contextual grounding. Insights from the previous phases of work, along with various theoretical and creative prompts (such as the use of fundamental human motives to generate ideas) were used to develop interventions. The quantity of ideas developed at this stage required a range of selection modes. Intuition was used to narrow large amounts of ideas to a more manageable set. This involved designers, WASH specialists and behavioural scientists, all of whom identified preferred ideas with some rationale. Later, additional methods were adopted that refined ideas through theory and context.

A theoretical grounding followed Behaviour Centred Design including an explicit Theory of Change for smaller number of concepts (and all subsequent concept iterations) together with a mapping of behaviour settings from data gathered in the Assess and Build phases and further synthesised at the start of the create phase. This provided a general user journey while mapping social norms, roles and human motives to the actors, props and infrastructure in the setting. This approach also successfully allowed designers, WASH specialists and behavioural scientists to discuss behaviour in context from multiple perspectives. The theory of change was used and refined throughout the work to provide thinking around why change should occur, any potential risks to the desired change taking place and how to inform specific features of the final designs. These theoretical tools were used throughout the generation and refinement of possible interventions.

Contextual grounding was achieved through the use of participatory design techniques wherever possible. This begins with grounding in the deep qualitative work in the previous two phases. After initial ideas were developed and narrowed, immediate feedback was sought in a focus group with several households in Tanzania. Further refinement of the concepts and subsequent prototyping allowed for detailed feedback from a second field visit to Tanzania. During these visits, specific questions were asked about the desirability, feasibility, viability and behavioural influence grounded in the theory of change. In-field changes were made to product concepts were possible based on feedback from potential users of the intervention. This allowed for rapid refinement of the idea and further development of the concept. Finally, a new concept was introduced that included a clear rationale regarding why it should work and enough conceptual definition to understand how it would be made.

#### 0 to 150 ideas

In a London-based workshop, ideas were generated using ‘How might we’ statements based on insights from the fieldwork as well as through the use of theoretical tools including ideation using different types of human motivation as levers for handwashing. Examples include motivations to wash through Play or Disgust, understood as a desire to interact in a way that develops mastery and avoid pathogen avoidance, respectively [17]. To rapidly generate numerous concepts with an effective means of judging their potential, a range of stakeholders were engaged with broad expertise: local soap sellers, design research experts, object fabrication experts, and end users. Following a review of the findings from the field, the team confirmed the user persona and design criteria; the ‘How might we’ statements were also refined.

#### 150 to 11 ideas

These resulting ideas were initially reduced through a cyclical process of elimination by grouping repeated or similar concepts. Some of the concepts also clearly required more of a service design intervention than a product driven approach; the team identified that in this case simulating the service would be more valuable than trying to make a product. Finally, ideas were examined against the overall design specifications that had been identified by the creative team (see Table 3 below).

**Table 3:** Design specification criteria.

Required	Optional (Desirable)
Aspirational	Tie-in to other services
Reliable	Complex life cycle
Visibly removed dirt	Multi-tier entry to market
Kill bacteria	Sense of getting clean
Feel clean / smell clean	Secure
Reliable (for habit formation)	Accessible for elderly and disabled users
Fast (< 10 seconds, all in)	Retrofitability
Integrated with scripted handwashing use	Bespoke usage
Intuitive use	Easy to clean
Easy route to acquisition	Creates place for handwashing with soap
Durable	Encourages 2 handed hand wash
Motivates hand washing	Hands free water flow
Easy user experience to maintain	
Minimise use of consumables	

Ideas were eliminated using expert intuition and experience, leading to 11 concepts being chosen for refinement.

#### 11 ideas to 5 concepts

These 11 ideas were then presented to a focus group in Tanzania. The focus group facilitator asked a panel of 4 women leading households in peri-urban Dar es Salaam to appraise the 11 resulting concepts based on graphical and oral descriptions of the ideas. Additionally, the facilitator also asked them to rank their 3 favourite ideas according to:

- The product you would most want to show your friends (to assess the potential use of Status and affiliation as motivational levers).
- The best concepts for teaching young children to wash their hands (to assess the potential use of Nurture as a motivational lever).
- The product they were most likely buy, and how they would pay for it (to assess the potential business case for such a product).

Based on results from the focus group, five concepts were selected.

#### 5 concepts to 3 prototypes

Through initial group discussion and from field research feedback, two main design directions were defined from the concepts and design sprint outcomes:

- General purpose products that are broadly desirable and make it easier to hand wash through convenience by discounting the (physical, cognitive) effort required, but which could be used for other tasks.
- Specialised products that can only be used for hand cleansing, and so may ensure that, if purchased, they will be used only for that purpose. However, these may be less attractive since they are single purpose products.

The theoretical argument that general purpose solutions would change behaviour alone was weaker than the argument for specialised design interventions. This is due to the fact that the former only cause a cognitive discount as opposed to a focused disruption to the post-defecation practices that were reported during early field trips. Also, a general purpose solution had no clear mental model for users that would stimulate hand washing. Consideration of these theoretical factors reduced the number of viable concepts to three.

Following ideation and concept selection, the priorities for the final design specification were also refined, to allow the design teams to complete the detailed design for functioning prototypes for field testing. Additional testing plans were also suggested. Referring back to the original design brief to “design a post-defecation hand cleaning system which encourages hand washing with soap,” it was decided to focus specifically on concepts that would directly address handwashing, which relegated some options. The main dimensions for comparison of the concepts were as follows:

- Behavioural scripts for use
- Sense of contamination
- Desirability
- Design engineering requirements
- Feasibility/business model

Prototypes were prepared and used in a second field visit in which one set of households were left with prototypes for a week.

This left only three concepts to take forward. Each of the final concepts consisted of illustrations, a short description, three unique selling points and a Theory of Change according to the BCD methodology (see example in Table 4).

**Table 4:** Example theory of change.

Intervention	Environment	Brain	Behaviour	Outcome
New form of soap delivery system	Placement of the innovation in home environment	New motivation to handwash due to neophilia and desire for social status	Wash hands after toilet use	Improvement in various health measures

#### 3 prototypes to 1 final design

Due to the complexity of real-world operating environments, further prototyping and testing was done in the field. The objective of the prototype field testing was to be able to clearly identify which concept has the biggest potential to encourage post-defecation hand cleansing whilst also being desirable, viable and feasible. From the concepts generated in the design sprint and further analysis of qualitative field work, a design specification for three products was generated. In order to quickly manufacture testable prototypes, rapid prototyping technologies such as 3D printing were used, and detailed manufacturing cost analysis was suspended. The key aim was to generate prototypes to elicit potential user’s mental models of what the design solutions were and how they would or would not use them in their lives. Within this was a focus on specifically generating feedback as it related to the Theory of Change for each concept. Where possible multiple copies of each design were made to test in the field, the aim being to get as many diverse reactions and opinions on the design prototypes so as to be able to better criticise and select the final concept for further development.

The three different prototypes were taken to 10 different homes similar to those in the initial round of in-depth qualitative field research for comparability. The first phase elicited users’ mental models in order to ascertain which sort of technology they preferred. Each household was then assigned a prototype to keep with them for a week. Before returning, the design researchers hacked prototypes by combining design features from the initial prototypes that triggered the most promising responses in the first phase. The second phase consisted of understanding the extent to which users actually engaged with the products.

Finally, these three concepts were assessed according to how desirable they would be from end users, how feasibly technical hurdles could be overcome, the viability of the associated business model and how likely they are to change behaviour. Desirability was assessed through the reactions of users in the field and how well they understood the designs. Included in this was excitement to show the product to others (and other indications of desire) and an attempt to script the use such that it was obvious how the product should be used. Feasibility was determined through an examination of manufacturing (including local manufacturing) capabilities at a reasonable cost and with consideration for potential maintenance. Likelihood to change behaviour was assessed through looking at the potential routines using behaviour setting theory, as well as evidence from the initial field testing. Business model viability was determined in part by the identification of local stakeholders to whom ownership of the project can be transferred to ensure the success of the project once the research team has departed.

Using these criteria and the experience of field testing of the prototypes, the team chose a final winner. Once selected, a testing plan for this winner was also devised by the design and behaviour change team.

### Deliver phase

The overall objective of the Delivery Phase was to get to a proof-of-concept intervention based on the results of the previous phases. This phase brings all elements together in a final design that can be tested with a set of potential users. The final concept was therefore developed to the level of a functional model and demonstrated in the previously identified contexts to validate results. In order to develop the final prototype, the final design implementation was defined alongside the parameters for the feasibility study.

Following the prototype selection, the research and design team jointly critiqued the final concept and defined what could be changed and refined given the timeline, budget and ideal feasibility trial. Once the limitations had been agreed, an updated product design specification was created so that the designers could begin detail design and fabrication. Fabrication was conducted in the UK.

### Evaluation phase

The overall objective of the Evaluation Phase was to measure the project outcomes against the stated aims. The first aim of this work was to create a desirable, feasible and viable solution that could scale. Desirability refers to the interest the final users have in the product. This includes matching needs and wants of the individual to specific features of the concept to make it appealing. This was evaluated through qualitative feedback in an iterative fashion. Technical feasibility refers to the ability to produce, use and maintain a product in context. This was evaluated through benchmarking against design requirements produced throughout the study and communication with local and international manufacturers. Financial viability was determined to be a product that could exist in the long term without required financial subsidy or ongoing logistical support outside of a typical supply chain. This excluded solutions that would rely on long-term charitable giving or that exceeded the budget of the end users. Attention was also given to the supply chain and the margin local sellers would need to make on any product sales. Prices for these were determined by benchmarking similar product supply chains, enquiring about household expenditure and creating a product which fits within this range.

The second, and most critical, aim was to increase post-defecation rates of hand washing with soap among the target demographic. To evaluate this, we launched a proof-of-concept trial for eight weeks with a total of eighteen households, nine households in Morogoro and nine in Dar es Salaam. Of those, six households in each region were given the intervention and three acted as control and were given bar soap and told this should be used for handwashing. All contact with the households was carried out by a local team from Tanzania consisting of soap sellers recruited for the study and project partners including one of the authors of this paper. The nature of the intervention was such that it required weekly refills and this was carried out by the local soap sellers. Soap sellers were trained on how to do this and provided with compensation for help in their weekly engagement of the project.

Handwashing behaviour is notoriously difficult to evaluate as it can be highly private but involves wide acceptance of what one ought to do; thus self-reported behaviour is often unreliable [21]. Our analysis employed four means of evaluation to develop a more accurate understanding of hand washing behaviour: questionnaires, soap seller reports, electronic monitoring and a debrief with the soap sellers.

Baseline and endline questionnaires were administered to all households from a local Tanzania team including local soap sellers and project partners. The baseline questionnaire gathered data about household demographics, sourcing and usage of water, handwashing after defecation, handwashing before eating and general questions about the colour, shape, size and other attributes of the product (intervention or control). Questions regarding handwashing included general use questions (e.g., location, whether the toilet is shared, water in the location), frequency of use (“How often do you wash your hands after defecating/before eating? Do you use soap?) and automaticity of the handwashing behaviour adapted from [22]. Endline questionnaires asked similar questions to understand any changes in demographics, self-reported handwashing behaviour and the perception of the intervention following use as well as a willingness to engage and use the product in the future. Notably, no households were asked to pay for the product during the trial.

Sellers were also used as informants about the utility and use of the product by households. This allowed for a few important things to occur. Each week when the soap sellers would visit the household, they would document the location of the soap, note the quantity of soap left, refill the soap, indicate a timestamp for the electronic measurement by spinning the spool, and evaluate the condition of the unit. To aid with this, each soap seller was given a phone with camera capability and weekly funds for image transfer. Each time the units were counted that were left, it provided a weekly consumption amount for the household. Together with the number of people in the household, this allowed for an estimate of number of uses per person per day. The other function of this protocol was to ensure the unit was properly reset for electronic measurement.

Each unit consisted of a soap wrapped in a spool around a core. The core of each unit included electronics that would record the time and number of spins each unit made. The purpose of this was to further record when units were used throughout the week. This could help determine, for instance, how many days a week the device was in used and patterns of use such as single piece taken or multiple pieces taken. It would also help assess a concern that soap tabs could be taken just before the soap would be refilled by the soap sellers as a way of showing desired results.

Finally, a debrief with the soap sellers involved in the study helped to understand their perception of the product and use in context. A variation of the spool design–Tear tabs–were placed in two kiosks wherein the sellers were asked to display and try to sell the soap. They reported on the reaction of people who saw the tabs in the shop, how much money they sold tabs for, any indication by customers in terms of how they would use the tab, how people carried the tabs once purchased and any other comments. The soap sellers who did refills (2x2 locations) were asked questions regarding how customers and users responded to the product and their perception of the potential for the product to sell.

Table 5 provides an overview of all the different methods used with households by ABCDE Phase.

**Table 5:** Outline of methods used by phase of study.

Phase	Objective	Methods	Results
*Assess*	Establish an overview of the latest understanding and interventions around handwashing with soap in low-income contexts, particularly Tanzania.	Literature review, market research and existing product testing.	Behavioural determinants, pre-existing products evaluated and a focused set of insights from local governmental, NGO and private industry, compiled to inform the plan for field research.
*Build*: In-depth qualitative field research	Develop a detailed understanding of the various elements of the behaviour settings of interest and the potential hindrances or affordances to the desired behaviour.	Behaviour observation across locations and times of day, individual and household interviews, and exercises/design probes to elicit information on psychological determinants of handwashing.	A database of qualitative information informing the design specification including: behaviour setting, user personas, local manufacturing and sales channels assessment. Insights and problem statements for idea generation.
*Create*: Concept and prototype development	Develop several effective conceptual offerings (i.e., interventions) that would be suitable for testing in context. A methodological goal was to merge human centred, behaviour centred and business model design methods within a single design process. Select the final concept to further develop for a feasibility trial.	The key steps involved concept generation through a design sprint, feedback from a focus group in Tanzania on initial concepts, refinement of concepts through a conceptual exploration of a theory of change and the fabrication of several prototypes were fabricated and tested in field. Frequent reflections within the team and documentation of comparisons between design processes, how they link and how they can be merged. Assessing the concept for potential to change behaviour, design thinking factors: desirability (contextually appropriate), feasibility (manufacturing) and viability (business model).	The successful completion of this task demonstrated a range of offerings and a measure of the feasibility, desirability, viability and potential to change behaviour with one being selected with a contextual understanding as to why it was the best concept. It also documents a new process combining the virtues of HCD, BCD and business modelling, providing a gauge of the process’s ability to produce useful products, as well as its general utility for other projects. Tab soap was chosen as the final concept to refine. In-field test demonstrated that it was easy to use for handwashing and nothing else. The individual units also offered a resilient business strategy.
*Deliver*: Final design development and implementation	Refine the final design based on in-field reflections and manufacture prototypes in order to test different hypotheses in the feasibility trial.	The design was refined through translating field observations to a product design specification. The protypes were made using 3d printing and novel manufacturing methods for the substrate.	Two products were designed and fabricated for the in-field trial. One was to be kept in home, preferably in the toilet with refills requiring a weekly/fortnightly update. The second was a tear-and-share designed for local kiosks to explore different sales channels and marketing opportunities.
*Evaluate*: Field trial	Assess the final design intervention based on its potential to change behaviour, design thinking factors: desirability (contextually appropriate), feasibility (manufacturing) and viability (business model).	A core field trial was devised using local soap sellers to distribute the product and routinely check in and remotely report usage to the design team. An additional sales trial was undertaken to understand preliminary attitudes to the novel handwashing product.	All participants used the soap during the trial for a recorded average usage of 1.48 tabs per person per day (mean SD = 0.69). Notably the participants showed continuous usage throughout the 2-month trial.

The research team also conducted daily sessions of thematic analysis as an in-field reflective session on the fieldwork findings. Team members would first write down key observations and insights individually and then cluster these into themes altogether. Insights and themes were transcribed and used in comparison to the overarching objectives to help adapt the subsequent day’s research plan. Every few days, the team would synthesise all findings into core opportunities for interventions framed as ‘How Might We’ statements that could be brought into the next stage of the study.

The following sections provide an overview of results by each phase. Finally, general discussion and conclusion sections summarise the implications of the study.

## Results

### Assess phase–contextual understanding

The objective of the literature review was to establish an overview of the latest understanding and interventions around handwashing with soap in low-income contexts with a particular focus on Tanzania. This informs the field work plan and the design team’s understanding of the hand washing in the context of design for behaviour change.

An overview understanding was achieved through a focused literature review and examination of the competitive landscape. The focused review considered reports of handwashing interventions especially those interventions that are product-based [23–25]. This provided some understanding of critically reviewed interventions and helped to inform aspects of the field work and design planning.

A total of 50 low-income hand wash products were identified through the literature review and internet searches. The search included some products developed for contexts related through low resource, low cost or other parameters such as refugee camps or holiday camping. These products were critically appraised according to desirability, feasibility and viability. The appraisal was based on an examination of available information online such as technical parameters, primary or secondary reviews and financial information. Five key categories of products emerged from the analysis: vernacular, Do it Yourself (DIY), child-focussed, soap-less wash and camping products. A short description of each category together with an illustrative photo and pros and cons are presented in Table 6. The critical assessment of the competitive landscape informs design specifications and fieldwork.

**Table 6:** Types of existing handwashing products.

	Vernacular	DIY	Child-focused	Soap-less wash	Camping
Type	Handwashing solutions that are not designed. These are usually pots and jugs of water.	Designs created to be made from upcycled and waste materials (e.g., tippy tap).	Handwashing products designed to attract and engage children.	Replacing traditional soap and water with one product, this also includes hand sanitiser.	Products that are made to be lightweight and portable for camping.
Pro	Easily available and inexpensive. Flexible use makes it broadly attractive to own and easy to adapt.	Very inexpensive and easy to maintain. Can be made with no bought resources.	Attractive for children. Early habit formation stays with child for life. Can also motivate parents.	Less money over time. Effective in drought-prone regions. Soap/cleanser built in.	Potential for complimentary markets. Super portable and generally easy to refill.
Con	Not specifically for hygiene so easy to contaminate. Does not clearly encourage or attract handwashing.	DIY quality is not very durable, and repairs can put people off from properly using equipment. Often seen as messy and low status.	Toy specifically for children could be seen as frivolous and unnecessary additional cost, less multipurpose.	Introducing new interactions is less intuitive and requires building new behaviours from scratch, risky.	Lack of permanence may not encourage behaviour in home, refill all the time is tedious; can be expensive.

A persona that represents a realistic target user of the end solution was developed through discussion with the stakeholders. The following User Persona was also developed:

*“Nana is a mother (head of family), who as well as cooking, laundering and cleaning house, needs to fetch water and buy and maintain all consumables in the house. Nana sometimes works selling either food she cooks, crops from local plot which she grows or small goods from the front of her house, by no means a stable income. Nana is expected to teach the kids and ensure that they wash their hands. She’s also sick of the in-laws passing judgment on her mediocre toilet facilities.”*

A review of existing background information was developed together with contextual parameters for design consideration. Stakeholders together represented some understanding of the daily context of the target users including economic, social and cultural dimensions. These contextual parameters included:

- No plumbed water in household
- Handwash inside the toilet
- Low income of < $6 a day (unskilled average)
- Soap budget of TZS1750/month
- Family toilet with concrete floor

From the literature and expert review, the key design priorities were defined as outlined in Table 7.

**Table 7:** Design priorities for a handwashing solution.

**Cost**	Unsurprisingly, in low-income contexts, money is a major barrier with regards to investment in sanitation products and infrastructure.
**Security**	Fancy products would not be left in or near the toilet for fear of theft.
**Usability**	Anything tricky or confusing to use would not be used.
**Hygienic**	Visual or perceived contamination of surfaces, space and soap is a barrier to handwashing with soap.
**Priority**	Designs would need to counter the observation that hand washing is the lowest priority usage of soap.
**Behaviour**	There is broad knowledge and understanding of the need to wash hands, therefore lack of education is not a barrier to handwashing with soap.
**Efficient with resources**	In households which carry in water, minimising the use of this precious resource is critical.
**Reliability & Maintenance**	In order to build habits and new patterns of behaviour sustained repetition of key behaviours is critical. Therefore, the solutions must work continuously and be easy to repair.

### Build phase—In-depth qualitative field research

**Fig 1:**
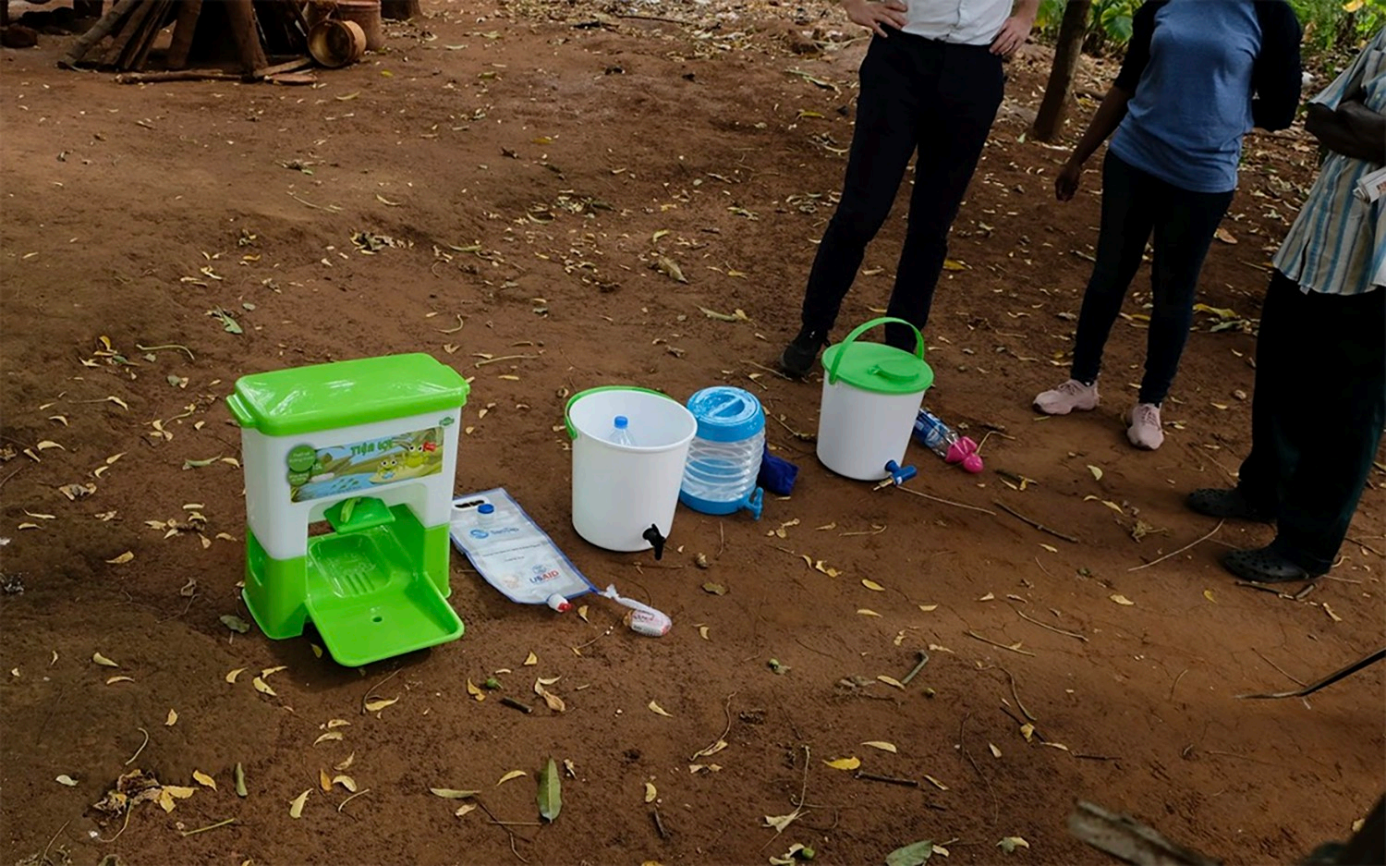
A research participant ranks the products by the most modern (left) to the least (right). (Left to right: Happy Tap, Sanitap, Oxfam bucket, Accordian, Supertowel, Oxfam + nagmagic, Spatap).

Several existing handwash technologies were taken to the field and tested in households for perceived feasibility, desirability, and viability (see Fig 1). Following initial interviews additional technologies were ‘hacked’ (e.g., from locally available products, such as turning a plastic table stand into a soap holder) and then used as stimuli in households to further explore the range of potential hand cleaning systems.

Critiques of these products were based on their functionality as aids to handwashing (e.g., whether one or two hands were needed to use it, how much water it used, etc.). At the beginning of the field work the products were presented and assessed individually. The products were ranked through the following categories.

- Most likely to show a guest
- Most durable
- Most modern
- Most exciting
- Most difficult to use

Following the first six household interviews, conversational ranking exercises were conducted helping participants better express differentiation between products and subsequently better understand preferences. The remainder of this section reports different product critiques and associated themes that emerged from the product ranking.

### Happy tap

The clear favourite was the Happy Tap, preferred by all as it was attractive, modern and it had the integrated capacity of storing water in a sealed unit. Some participants also referred to the benefits of using it to teach their children how to wash their hands, citing the motivation afforded by the bright cartoons on the packaging (MG3). The only concern mentioned was that the sprinkler tap did not provide a satisfactory flow of water (MG3).

#### Sanitap

The Sanitap bag had mixed perceptions regarding the user experience: MG2 found the unit confusing, yet some children found the graphics on the back clear and easy to follow. The soap attached in a net was seen as a key benefit as it acted as a reminder to use soap to wash hands. Being easy to hang off the ground was seen as another benefit, preventing the unit from getting contaminated. Some participants also cited a perceived lack of durability as the unit was a plastic bag, saying that it would not be kid safe.

#### Oxfam bucket (& Nagmagic tap)

The Oxfam bucket was simple to understand and generally perceived as a very useful and durable object. However, it did not communicate handwashing clearly. As MG1 said, ‘Honestly, [I] will use it for drinking water’. Other households made similar comments around the bucket being attractive for drinking, laundry, washing dishes or other activities (DSR1, DSS1, MWR2).

#### Spatap

The Spatap was one of the least popular solutions as the unit was perceived to have too small a capacity for hand washing (MG3). A sense that the rubber would be easy to break (MG3), despite being made from the most durable material was also mentioned; perceptions of durability were linked to the potential to purchase. A positive comment was that the water was enclosed so that it would not be contaminated by the air in the toilet (DSR4).

#### Supertowel

The Supertowel was met with comments such as ‘If it is true, it simplifies things’ (MWS14) and ‘Its fake’ (MWR12) when respondents were prompted for appraisal, indicating a tricky mental model. The fact that the Supertowel’s immediate use was not apparent was an issue. As there was no comparable product, the participants suggested different benefits: MG1 would share it with the entire family whereas the son at MWS14 said he would take it when travelling on long journeys and use it to wipe sweat from his head, not for handwashing. It was seen as desirable for being a modern technology.

#### General observations from product ranking exercises

Ease of use for handwashing with soap meant having a reliable system that facilitated the current mental model that handwashing is a two-handed job. This means solutions that are better allow for ‘proper’ hand washing which ‘requires two hands’ (MG4). Presence of soap (i.e., Sanitap mesh, Happy Tap spot) acted as a reminder. Refilling often is not convenient, the Happy Tap’s large storage capacity in this respect was seen as a benefit.

One of the key benefits of the ranking exercises and using existing products for interviews was that it required participants to verbally relay their initial interpretations of the objects presented to them. The aforementioned Supertowel, while an excellent and well-received product was hard to understand. The Sanitap unit had a similar issue: being a novel product made it hard for participants to know or imagine what it could be used for. However, the Sanitap solved this issue by having a clear set of pictographic instructions on the back. The Happy Tap was particularly successful in this regard as it includes a large bay that is clearly used for a bar of soap.

Another issue, common to the Sanitap, Oxfam bucket, and Spatap, were that respondents said that they would use the units for storing drinking water or food, not handwashing:

- “[I] can use Sanitap for drinking as long as it doesn’t go in the toilet” (MG1)
- Oxfam bucket would be used for storing drinking water (MG1)
- Spatap would use for drinking (MG2) and showering (DSR4)

This is linked to a key observation that handwashing is the lowest priority usage of water and soap, meaning that products will simply be used for other activities. Most households had either no soap or dry slivers present in the toilet indicating that there were no soap products for handwashing. The buckets for cleansing were usually old storage buckets that were close to breaking point.

#### Phase summary

From the daily thematic analysis sessions, the team highlighted 75 different topics through the lens of handwashing and the products, services and systems that facilitated the behaviour. Themes evolved over time. For instance, as some topics emerged previously, but became clarified, the team would shift focus to areas that were less well understood. Each theme highlighted a potential design priority, a topic to address when generating ideas and solutions. The most pertinent themes and observations are addressed below.

All participants expressed knowledge or understanding that one should wash their hands with soap after defecation. Throughout the household visits, the team enquired as to how families sourced water for various activities (cooking, cleaning, bathing, dishes, laundry, etc.) and the different soaps used for each. Clean water would be kept for drinking, cooking and potentially clothes washing. Water for handwashing was commonly the same water used for flushing and wiping in the toilet. Soap usage had a similar linear downward trend, with fresh bars of soap being reserved for body washing, then clothes and soap slivers sometimes being used for handwashing. While these were often kept in the toilet, it was clear that many were hardly used once they became so small with participants sometimes not knowing the slivers were in the toilet. Liquid soap was generally used for dishwashing or cleaning a floor but was less commonly found in the homes. Powder soap was used for washing clothes.

A sense of contamination was an evident barrier to handwashing with soap and took on many different dimensions throughout all visits. The target of contamination included just about anything in the toilet area (the soap itself, the air, floor, walls, objects brought into the toilet, etc.). Interestingly, in the case of soap, even an object meant to be clean and to clean dirty things it comes in contact with, was perceived to be contaminated. The sense of contamination around sharing soap and the usage of water presented two very clear design challenges in being able to keep products near the toilet while assuring individuals that no cross contamination occurs.

### Create Phase–Iterative concept and prototype design

#### 0 to 150 ideas

**Fig 2:**
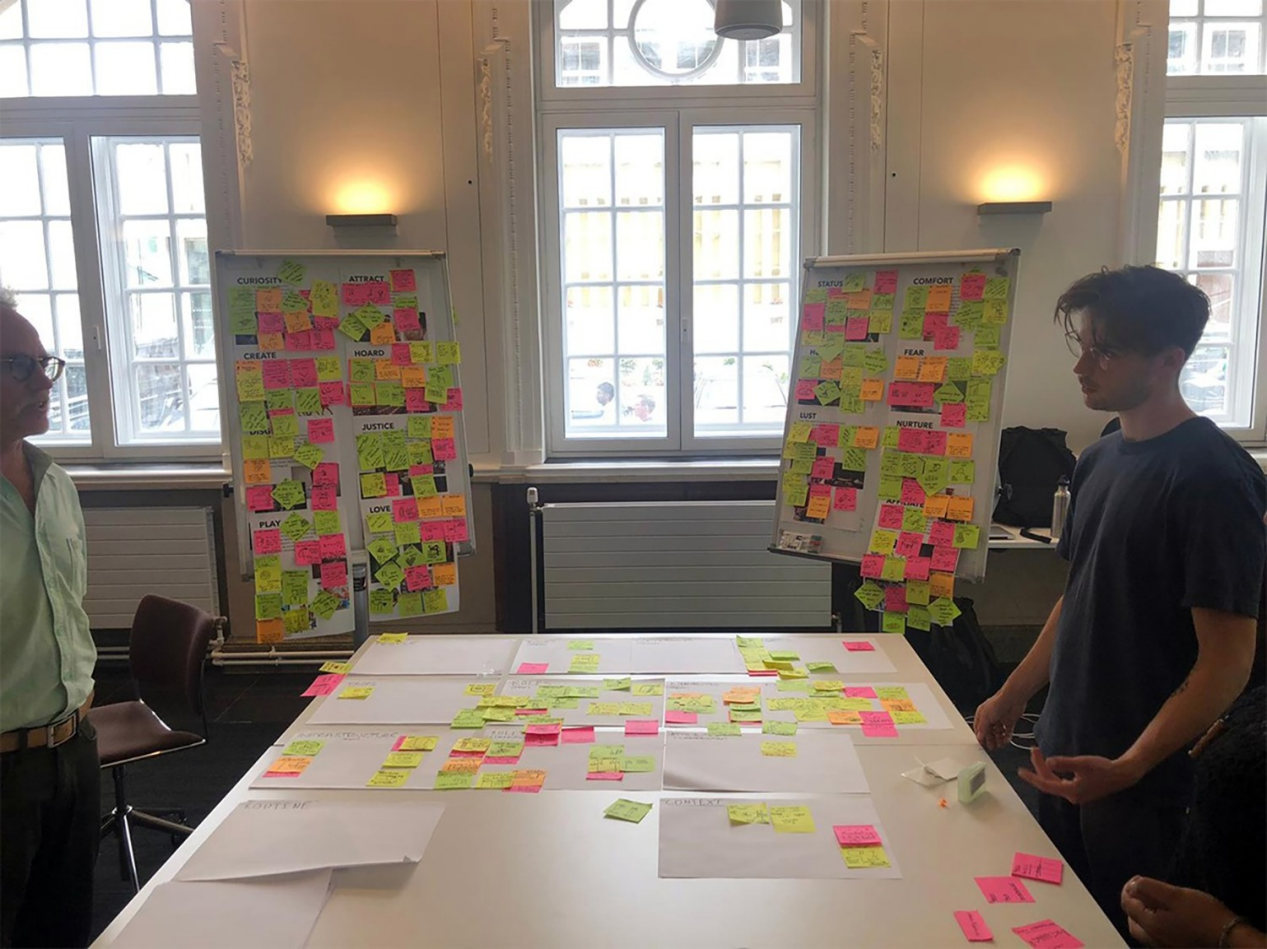
An image of an idea generation activity within the design sprint.

The London-based ‘design sprint’ workshop generated more than 150 ideas from ‘How might we’ statements and other provocations, captured using post-it notes (see Fig 2).

#### 150 to 11 ideas

A variety of clustering and expert ranking exercises during the Design Sprint Workshop reduced the plethora of initial ideas down to 11. Clustering activities included consideration of the type of motive(s) embodied in the intervention and the type of intervention made (e.g., product, infrastructure, service). Ranking considered opinion around technical feasibility, a viable business model and some intuition around how desirable the idea would be or how likely it would be to work (i.e. influence behaviour).

#### 11 ideas to 5 concepts

**Fig 3:**
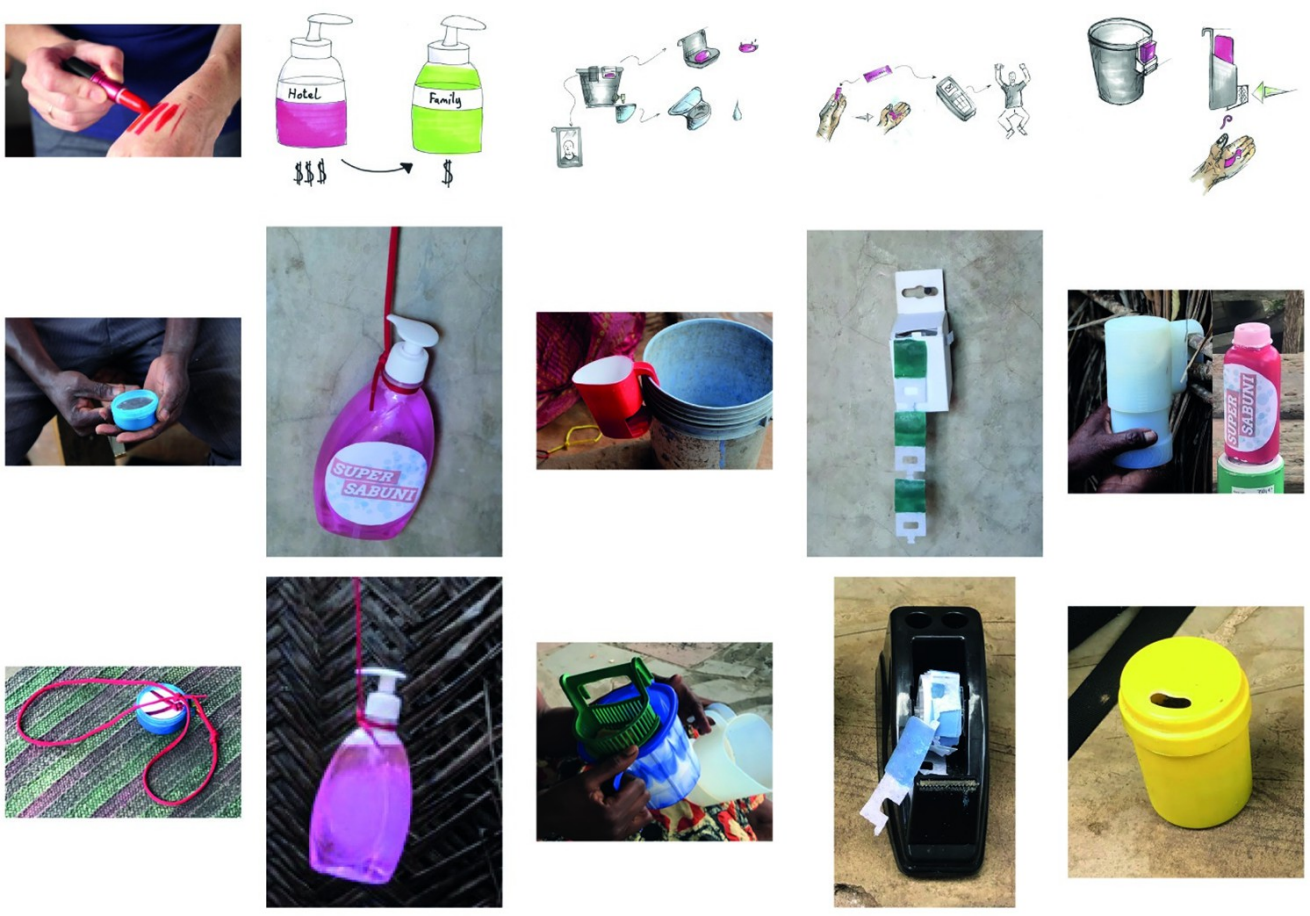
An overview of the development of 5 chosen concepts as initially sketched (top), physically prototyped (middle) and adapted in field (bottom). From left to right, concepts are Personal Soap, Liquid Soap, Cup, Tab Soap, Soap Shaker/Grater.

A focus group in Tanzania run immediately following the idea generation helped to make it clear which of the 11 ideas should be further developed. Subsequent concept development and selection stages are described below in Table 8 and illustrated in Fig 3. One major variation was made from the Tab Soap concept. Initially, the concept was thought to include writing hidden under the soap which would reveal a potential win. Randomised prizes proved very difficult to manufacture which increased scepticism relative to its potential impact. The prototype of this concept therefore consisted solely of a rectangular fabric substrate impregnated with soap.

**Table 8:** Concept development matrix following each of the five concepts from their inception in the design sprint through to the end of the prototype field testing.

Concept Name	Personal Soap	Liquid Soap	Cup	Tab Soap	Soap Grater/Shaker
**Concept description**	Personal soap dispenser can be carried in clothing. Simply extend soap by twisting bottom of dispenser, rub on hands, foam up with water. For use anywhere.	Seller collects used premium hotel soap bottles, fills with cheap liquid soap for sale to households. Can be refilled from local kiosks.	Existing (i.e., used) buckets and cups are modified. The modification may be a retrofit service. One accessory would be a storage place for soap in existing cups.	A single use soap for handwashing. Each tab has a code that you can message using your phone. The message enters you in a chance to win a prize. If you enter every day, your chances improve.	When you push on the device, it dispenses a slice of soap appropriate for single use which is grated from a bar of soap. Any kind of soap can be used in the device.
**Modification from field feedback**	A strap was added to aid in carrying the soap and using the soap in various contexts where hands may need to be free without placing this in a pocket or on the ground.	It was found that door to door liquid soap sellers were in operation which indicated the feasibility of a liquid soap initiative in the city. However, finding a reliable source of recycled bottles proved challenging.	The soap storage was modified as the current design’s soap storage was not immediately clear, nor was it effective for storing soap when being dipped into the bucket.	The amount of soap was deemed too high, therefore the tabs were modified. The box was too flimsy so a more solid design from a repourposed tape dispensor was used to indicate a superior product. The idea of dropping the prize was also suggested.	Although the grater was kept for testing, it was deemed ineffective and adapted as a shaker. The Soap shaker was modified to accommodate soap flakes which generated a lather more similar to soap bars over soap detergant.

#### 5 concepts to 3 prototypes

The second field visit utilised prototypes (Fig 3) of the five concepts to gather feedback and evaluate an understanding of success factors including the potential to change behaviour. While some households used these prototypes for a week, other households provided concept rankings during a rapid visit. The combination of these kinds of feedback resulted in two concepts—the soap grater and personal soap—being eliminated. The soap grater was too complicated to use, requiring too many additional cognitive and physical steps in the handwash routine. The unit would also be too expensive, requiring a bulky product and custom soap. Throughout the household visits no indication was given by participants that it would be intuitively used for handwashing over body wash. The personal soap was seen as desirable but wasn’t in fact ever used (for reasons difficult to ascertain). It proved very difficult to procure recycled soap bottles in Dar es Salaam, so there didn’t seem to be any business model associated with that prototype. The most used prototype was the tab soap. There was also real enthusiasm expressed about the possibility of being able to have such a product available in the local market.

Households were also asked to rank the five concepts based on various desirability indicators (Table 9) and likelihood to use for handwashing (Table 10). Some indicators of desirability seemed to successfully reflect the perceived quality of the prototype, usefulness of the product and familiarity. This is true for the liquid soap and cup in particular. However, there is some question of whether this would be used for handwashing or if it was desirable in its current form. Tab soap was clearly seen as the most modern and equally seen to be useful for handwashing.

**Table 9:** Prototype field visit ranking exercise.

Ranking category	Preferred prototype (Number of households)
**Best to show off to neighbour**	Liquid Soap (4); Soap Grater (4)
**Best to teach children handwashing**	Cup (4), Liquid Soap (3)
**Most modern**	Tab Soap (4)
**Most likely to buy**	Cup (4), Tab Soap (3)
**Favourite prototype**	Liquid Soap (4)

**Table 10:** Household rankings for prototypes that were most likely to be used for handwashing.

Household	1	2	3
**HH5 daughter**	Liquid Soap	Tab Soap	Personal Soap
**HH5 mother**	Tab Soap	Personal Soap	Liquid Soap
**HH1**	Soap Shaker	Tab Soap	-
**HH2**	Tab Soap	-	-
**HH8**	Soap Shaker	Tab Soap	-
**HH7 husband**	Soap Shaker	Cup	Personal Soap
**HH7 wife 1**	Soap Shaker	Tab Soap	-
**HH6 husband**	Tab Soap	Soap Shaker	-
**HH6 wife**	Soap Shaker	Tab Soap	-
**HH9 elderly**	Cup	Soap Shaker	Personal Soap
**HH10**	Tab Soap	Soap Shaker	-

#### 3 prototypes to 1 finalist

The choice between both design approaches is non-trivial as selling specialised products is less feasible (the target demographic observed in our research showed that they primarily owned multi-function products). The idea of selling a multipurpose product that has nudges to encourage hand washing with soap seems like an easier product to sell and was reflected in the HH rankings (see Table 9) where the cup was the most likely product to be purchased.

The most pertinent points of comparison for each prototype are listed below (see Table 11).

**Table 11:** Major reflections on 3 final prototypes.

Tab Soap	Liquid Soap	Shaker Soap
**Positive** Tab Soap could be used through 2 routines. The more diverse the use cases the better. The Tab Soap can be kept in the home or the toilet and the container needn’t move. A proprietary manufacturing process would make the sales and development more economically attractive to local entrepreneurs and designers. **Positive & Negative** Completely new handwashing paradigm: if we are elevating handwashing, then a novel product could establish a new mental model for handwashing. This could be negative as mental model is more complicated and needs to be established.	**Negative** Recycled bottles require cleaning and possible repair. Due to the naturally disposable nature of these bottles, economics of cleaning and repairing bottles for resale is unclear. Compared to Tab Soap, this was still less likely to be used exclusively for handwshing due to a persistent mental model of liquid soap for washing floors as observed through field work. Cost optimisation on the soap bottles is not possible, would need to redesign from scratch or run a different business model. **Positive & Negative** Generic refills make the business model more vulnerable to competition and therefore potentially less desirable to salesman. This could be positive as there is already an established market with corresponding sales channels.	**Negative** Not as desirable. One key part of the new product needs to be desirable and this plays into soap being modern as well. Would require a handwashing specific powder to be developed; it would need to usurp the current mental model that powder soap, while effective, is low status. Powder Soap leaves hands feeling slightly greasy and dries them out as well To dispense soap, the container needs to be picked up off the ground adding an additional step to the routine of post defecation handwash when compared to the other solutions. **Positive & Negative** Generic refills make the business model more vulnerable to competition and therefore potentially less desirable to salesman. This could be positive as there is already an established market with corresponding sales channels.

#### Final design concept: Tab soap

An ideal process would afford further exploration of all concepts to address shortcomings and test more broadly. However, resources and time constraints required that we make a selection based on expert intuition. The Tab Soap was selected on this basis, as well as the fact that it had been the only prototype that had been regularly used by the testing household, since this criterion was the *sine qua non* of a behaviour change project.

The final design of tab soap was articulated according to unique selling propositions which would later be further tested in a proof-of-concept study. This was articulated as follows: For low-income residents who want to leave cholera behind and become modern, *Sabom* (Swahili for soap) is soap for handwashing that gives you a fresh piece of soap every time you use it, even in a shared context. This contrasts with the common bar of soap found in the toilet which is seen as disgusting.

Key unique selling propositions:

- A soap dispensing solution that delivers individual soap tabs
- Each serving can be easily transported from dispenser unit to location of use
- Each serving can be temporarily stored and carried and it remains durable
- The servings have just enough soap for handwashing, making it hard to use them for other activities, a core insight was that if soap can be used for other activities then it will always sideline handwashing with soap
- The dispenser unit holds enough to serve a family for 1 week (preferable for habit formation)
- Units can be sold on an individual basis, allowing for flexible price points and quantities to help sellers find the most suitable price point to encourage customers with reduced means to start using the product
- The soap can’t be used easily for other purposes, so when you give them to your family you know what they’re doing
- The form factor allows for significant behaviour change oriented messaging

**Fig 4:**
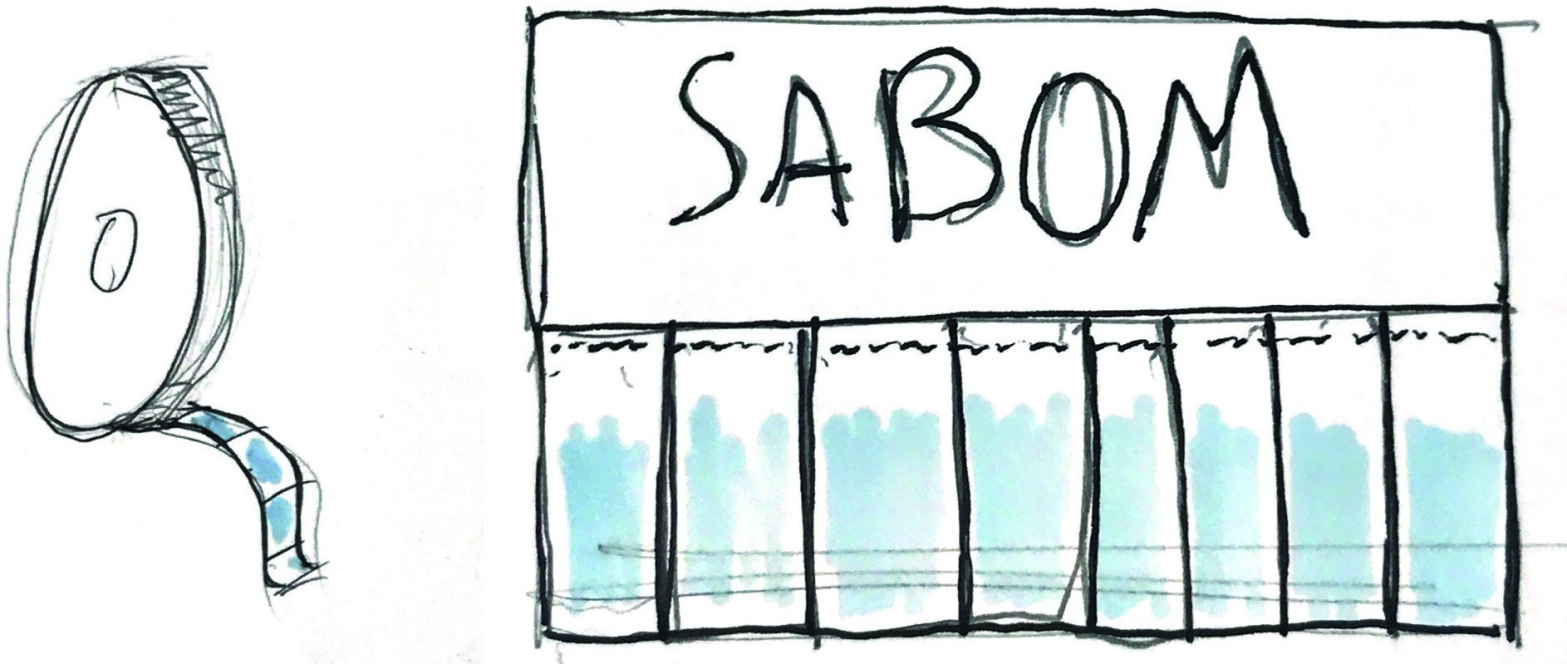
Sketches of the roll (left) and the stacked tear and share (right) form factors of Tab Soap.

Several form factors were considered and two were developed for testing. Table 12 describes the two form factors which are visualised in Fig 4.

**Table 12:** Tab soap form factor analysis.

**Rolls**	**Stacked tear-and-share**
The dominant format considered is selling in the form of rolls–the idea here being that the tab soap can fit into more substantial casing designs to provide long term solutions inside people’s homes as well as being convenient for soap sellers to distribute as they wish.	A second format comes as tear sheets. The benefit is much larger real estate for advertising and hygiene-based messaging. In a shop context it will also provide a visually engaging prop.
**Pros / Cons**	
• More aspirational form factor • Contamination protection (fully sealed; only one tab exposed at a time) • Can fit into plastic casing or stand alone • Casing provides water resistance • If unit is lost in dispenser, hard to find missing tab	• No need for casing (or plastic) and thus very low cost • Can be used as effective display in shop • Easy to see how many tabs left • Packing can be quickly destroyed if get damp • Easily susceptible to water damage • Less novel and attractive

### Deliver phase–Implementation

The Tab Soap prototypes needed to be turned into working models for use in a field trial for evaluation of the product’s real utility and value. The main criteria feeding the finalization of the product design are listed in Table 13.

**Table 13:** Design criteria for the tab soap.

Criterion	Description
**Ergonomic experience**	User experience is critical in making the behaviour as easy and intuitive as possible. The products need to be usable by people who may never have seen or dealt with a new product before. This included the delivery and use of the soap with considerations such as optimised soap application and balancing quantities used.
**Easy to fabricate**	Design features were modified in order to allow for final design to be rapidly manufactured which required compromises in shape and material. Consideration was made for sourcing materials and various material considerations such as suitability (biodegradable, cheap, balancing comfort and structural integrity)
**Contextually suitable**	Although not a focus, the aesthetics and labelling of the product was defined using behaviour setting theory and aesthetics were presented through fixers in the field to ensure contextual suitability.
**Within household budget**	Initial cost estimates were produced to make sure that the product had a reasonable price range and to use them to probe potential soap sellers as to whether they believed in the market potential of the product.
**Measurable**	The concept would need some features to aid the evaluation of the behaviour in context.

The final design was refined based on the above and formalised with a product design specification–a document used by designers that specifies different user requirements and links them to specific features to be created. In line with the development of a hybrid design and behavioural approach, the behavioural determinants from the Assess stage were used to prioritise different features. A Theory of Change developed for the products at the prototype phase was also used to compare and highlight important design features for the team.

### Material selection and manufacture for the feasibility study

During the detail design phase, material selection was a key step as it has a direct impact on the manufacturing process with direct consequences onto the feasibility trial size. The process began with the sourcing of numerous alternative candidate materials for the final design. They were then tested and selected based on a balance of user experience and manufacturing speed. The manufacturing process was designed taking into account the potential tools and workforce available in Tanzania. When running small production runs for feasibility trials, access to realistic commercial materials is not always feasible; this means that compromise materials are sometimes selected.

**Fig 5:**
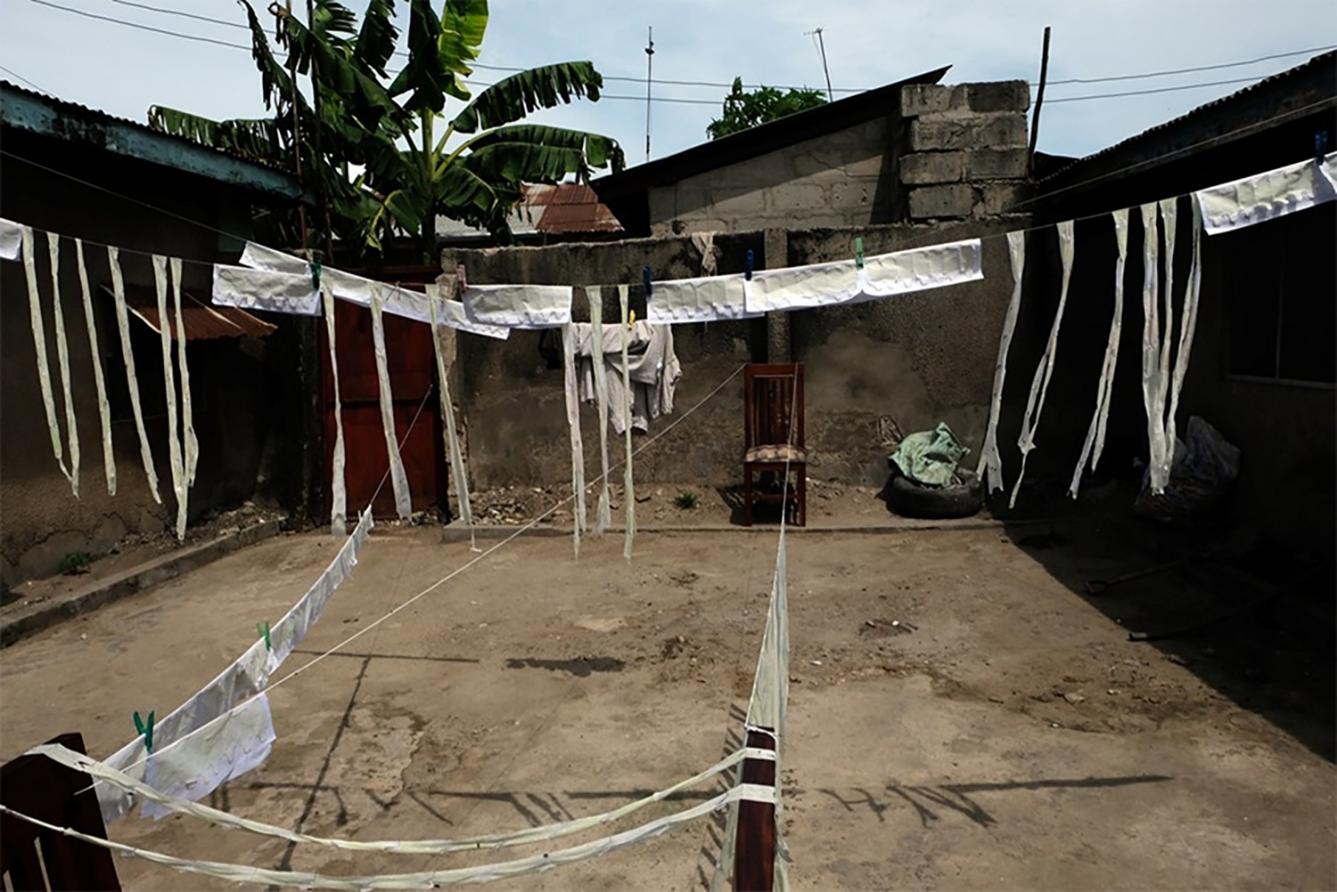
Drying of hand-made tab soap strips that were used for the field trial.

Given that the final product involves tabs of soap with a substrate of material, use of the product will produce small pieces of rubbish. A biodegradable material was chosen which decomposes in the pit latrines common to this type of environment. The material chosen was a bamboo-based textile which could be impregnated with various types of soap. Production of test samples of Tab Soap involved ad hoc manual methods due to the small scale of the trial (see Fig 5).

**Fig 6:**
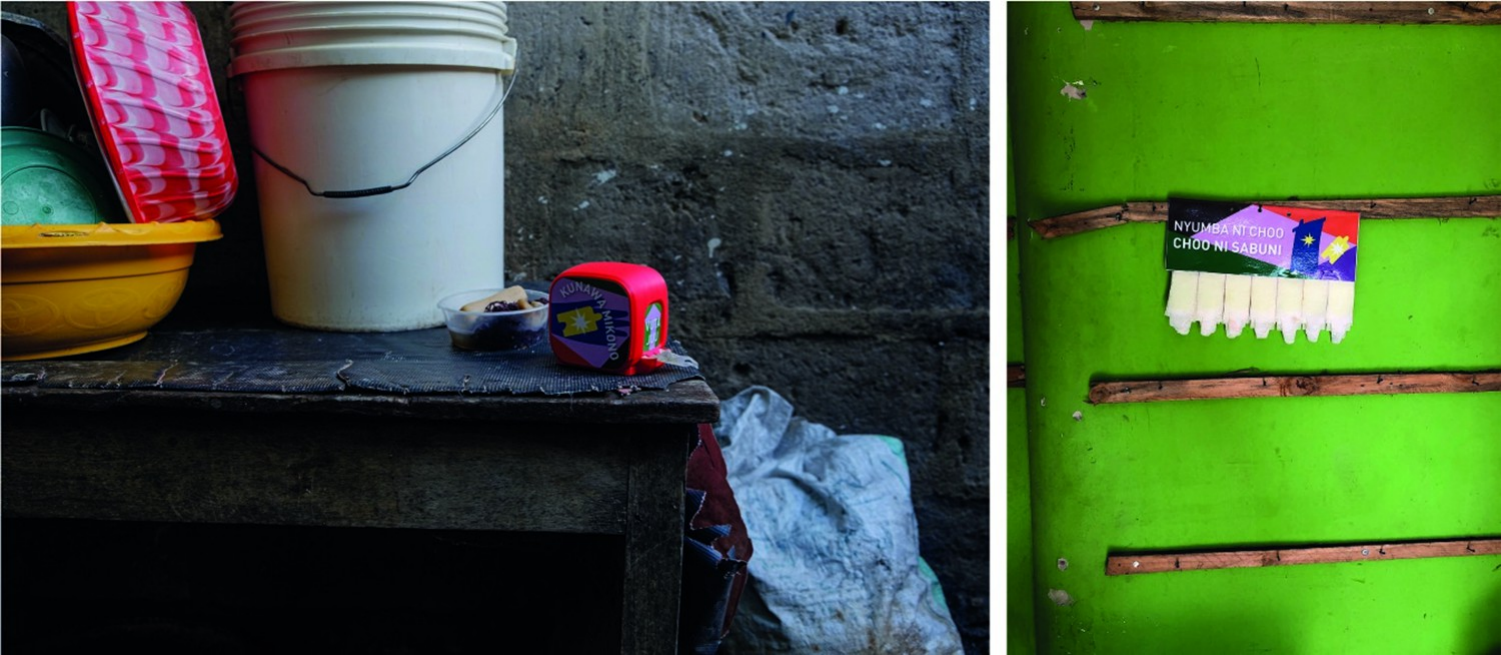
Tab soap form factors *in situ* during field trial. The left image shows the roll soap within a home. This version can be hung (e.g., on a nail) or placed on a counter. The right image shows the tear-and-share version mounted on a nail in a shop.

The two final designs (see Fig 6) for the Tab Soap produced offer a number of benefits. Both can be mounted on a nail which makes them visual and usable with a single hand. They use a biodegradable substrate and common soap making them safe and without need for clinical trials. The soap-impregnated substrate is durable and can be held in a pocket and carried around for weeks so long as it does not get significantly wet. A single unit was designed to last a household between one and two weeks.

In the proof-of-concept test, households were given the roll units. This decision reflects a core objective of seeing if the product would create behaviour change. The main interest was then around understanding if handwashing would increase with access to the product. While a detailed exploration of the business model is beyond the scope of this work, this was tested slightly through two avenues. First, the households were asked if they would buy the roll after they had used the product. This attempted to simulate a free sample model after which one could continue with purchases. Second, the tear-and-share tabs (Fig 6, right) were placed in kiosks and shop keepers were asked to try to sell these and gather feedback on how this occurs.

### Evaluate phase

Several kinds of tests were initiated to evaluate the reception and use of the final design, tab soap, in Tanzanian households from Morogoro (MG) and Dar es Salaam (DS). For instance, a household from Morogo will be marked as MGHH and the number of the participating household.

#### Analysis of endline interview data

Primary among these was an in-home trial. Feedback from the trial was generally very good. Everyone liked the soap: it was pleasant on the skin but did not smell too much, it made a lot of foam, and washed off easily with little water. ‘It cleans the hands well and it does not leave it with strong odour that will keep disturbing you later.’ [MGHH9]

The tabs were liked as well, and the novelty was generally seen in a positive way: ‘All [my visitors were] amazed and liked it. Paper turning into soap is not something we are used to.’ [MGHH4] They liked the portability too: ‘I would put them in my handbag and take a few and put [them] on the table’ [MGHH4]. This act of collecting tabs and putting around the house (e.g., stored outside of the device) was repeated in other households to keep out of reach of children or just to be more convenient.

The dispenser was seen as good-looking [MGHH2], and as protecting the soap inside from weather and insects. This led to a consistent and important report of no contamination. ‘Yes, I like it very much. […] The tabs are good and prevent transmission of diseases to others. The tabs are covered and insects can’t lick them so it remains clean and uncontaminated.’ [MGHH2]

However, the mechanism did not work perfectly as sometimes households pulled off tabs without the next one coming out, requiring them to open the container up to fetch the next tab. In one case, the internal wheel did not spin well after refilling. [MGHH2] These experiences imply the container may be difficult to manufacture reliably at the necessary price-point. Even some of those with the container thought it might be better to just have tear-and-share.

The dispenser was kept in a wide variety of places, both inside and outside the toilet. One informant noted that the soap itself can serve as a stimulus to handwashing: ‘Yes, I like the soap. It reminds me to wash my hands whenever I use the toilet, unlike the bar soap.’ [MGHH3] This provides evidence for the Theory of Change successfully working.

Many of the households appear to have used the soap, often quite regularly. Some of the behavioural demonstrations suggested practiced use, others less so (or perhaps nervousness at having to be filmed while doing a behaviour in their toilet). A number of households reported running out of tabs before refills were made available, suggesting regular use. About half of the households claimed that they finished the roll of tabs before soap sellers came back with weekly refills. Expectedly, many of these were from households with a larger number of members which also led to the related smaller number of soap usage per day.

No one had difficulty disposing of the substrate, either in the toilet or throwing onto a trash heap after use. Several households had the idea to throw away the substrate before it lathered up too much, controlling the amount of soap they used from each tab (and thus the amount of water needed to wash it off) [Handwash demonstrations].

The system was definitely something newsworthy in the neighbourhood. Every household mentioned showing the container to neighbours or visitors. ‘When a visitor goes to the toilet, I wait for them outside because I know they will ask me about the container. Then I teach them how to use it.’ [MGHH2]

Users prefer to have relatively large quantities of tabs on hand, as they fear the uncertainty of visitors coming and needing soap, and don’t want to repeatedly have to go somewhere to purchase tabs, which they find annoying. The price is always a worry, but they seem to fear not having soap more. Even though the dispenser was seen as large, some wanted it to be even bigger to hold more tabs. [MGHH2] ‘I always want them [my guests] to use the tabs after using the toilet. I give them a tab use and tell them I can’t put them [the tabs] in the toilet because there is no roof.’ [MGHH4]

The fact that the tab soap was designed to be used only for handwashing purposes frustrated some users. ‘No…I will go back to normal soaps because the tabs are not for multi-usage. Other than handwashing, I cannot use them for anything else.’ [MGHH1] Several households mention trying to use multiple tabs to do other jobs, like bathing [DSHH6, DSHH8, MGHH5], washing hair [MGHH7] or clothes [MGHH5], but mostly as a consequence of not having other soap available. The experiences were also not satisfactory.

While novel, most indicated that it was easy to learn how to use, once you explained that it was paper that turned into soap. ‘[My elderly] mother taught us. It was okay, it’s pretty simple to use.’[MGHH3]

When asked for pricing, sellers mentioned prices considerably above the break-even costs though it is not clear if this is within the household’s budget.

Unsurprisingly, the baseline and endline questionnaires did not show a significant change in reported automaticity of behaviour. This is likely due to the novelty of the device and associated behaviour, and potentially changing circumstances of use, as households grew more used to having the product around the house.

#### Weekly seller visit data

**Fig 7:**
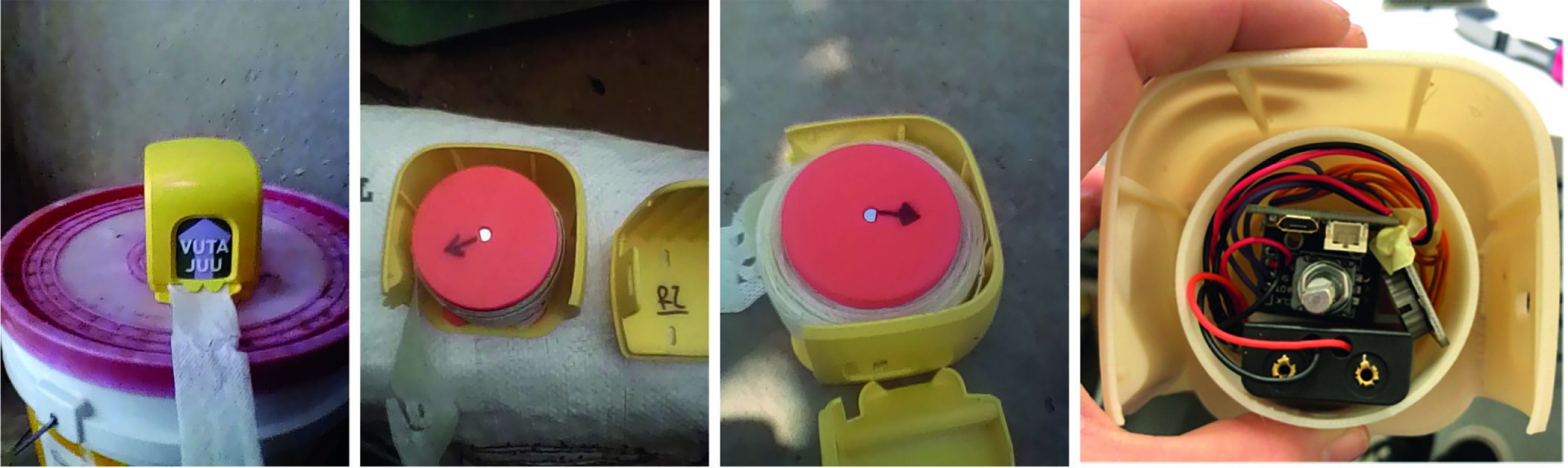
Example of weekly images from the soap seller. Images here show (from left to right): the roll dispenser, the remaining soap on the spool, the new roll of soap wrapped around the spool and the electronics within the spool.

The soap seller refill was documented through images gathered each week (see Fig 7) and through calls or sms to provide data indicating the number of uses during the week. The frequency and clear documentation in this method provides a more reliable data source for usage (see Table 14). The overall average use was 1.48 tabs per person per day (mean SD = 0.69). The number of uses was truncated in some cases where a household ran out of soap before the week was through. What stands out from a behavioural perspective is that all households continued to use the soap throughout the two months of the trial, and that while there was variation between household levels, the average use is as high as would be expected, given that the soap was often fixed inside the toilet and people were instructed to use it after defecation. We cannot tell from the data what proportion of household members used the soap, but there is anecdotal information in the qualitative data that it was not just one or two members, or that use was restricted to adults. Indeed, it was also extended to visitors.

**Table 14:** Soap use per household member each week.

Household code		1DS	1MG	2DS	2MG	3DS	3MG	4DS	4MG	6DS	7MG	8DS	9MG
HH members		7	3	6	3	10	3	2	3	5	7	9	7
Week/tabs per person per day	1	1.29	2.95	1.45	2.95	0.69	3.00	0.86	0.57	1.71	1.29	0.63	1.29
	2	0.82	2.05	1.45	3.00	0.51	0.67	4.50	0.71	1.80	0.84	0.13	1.27
	3	1.08	2.90	1.50	3.00	0.90	3.00	0.79	1.57	1.80	1.29	0.83	1.27
	4	1.29	0.71	1.50	3.00	0.90	0.00	0.57	1.67	1.51	1.29	0.00	1.12
	5	1.02	1.71	1.50	3.00	0.03	3.00	0.07	1.19	1.49	1.29	1.00	1.10
	6	0.82	2.05	1.50	3.00	0.76	2.00	0.86	1.38	1.74	1.29	0.52	1.22
	7	1.29	2.57	1.50	2.95	0.89	2.48	2.07	1.29	1.80	1.29	0.46	1.12
	8	1.16	2.90	0.79	3.00	0.90	2.76	1.43	1.33	1.80	1.04	0.38	1.10
**HH ave**		**1.09**	**2.23**	**1.40**	**2.99**	**0.70**	**2.11**	**1.39**	**1.21**	**1.71**	**1.20**	**0.49**	**1.19**
HH SD		0.15	0.26	0.17	0.01	0.34	0.39	0.31	0.13	0.07	0.13	0.33	0.06

Tab numbers are measured as per person per day for each household (HH). Household numbers across the top correspond to two locations: Dar es Salaam (DS) and Morogoro (MG). The missing numbers reflects the absence of those households who were given regular soap as a means of comparing.

#### Analysis of sensor data

The electronic sensor embedded in each device recorded the time of use and the rotation of the device (an indication of quantity used). However, the electronics were subject to dropping, water damage and faulty wires. The sensor also required a battery being changed halfway through the trial. The result was that data collected was limited due to various failures. Specifically, only nine of the twelve devices provided enough data to indicate some form of interaction. Still, the data (S1 File) corroborate the observation that tabs were taken from the units consistently throughout the week. Specifically, the devices were used on 112 days of 190 total operational days (those days when the device was tracking data) for a rate of 4.1 usage days per week. Critically, all devices which worked following a battery refill during week 6 also recorded ongoing use throughout the week, suggesting that use was consistent even at the trial end. A lack of activity may not mean a lack of use. For instance, the endline interviews indicated that people tend to take multiple tabs out of the device at once and store them around the house or on their person which they could use as needed without employing the device with the electronics, which would suggest that greater utilisation was had throughout the week on days when the device did not show activity.

#### Soap seller debrief

The response from the sellers was broadly positive. Beyond all having tried the tabs themselves, both groups of sellers in Morogoro and Dar Es Salaam agreed that they would be able to sell units and tabs following the trial. All the soap sellers managed to sell soap initially, however only the kiosk in Morogoro kept a repeat customer. The unit price settled to TZS 1500–2000, but these units included quite a few tabs. In Dar Es Salaam tabs were sold instead in smaller units, with one seller selling 4 tabs for TZS100 and the other selling 1 tab for TZS100. The Morogoro soap sellers all agreed that “there was no challenge on the price”.

All the sellers mentioned that without marketing materials they could not sell the soap as customers were not used to a product specifically used for handwashing, let alone paying for one. They stated that this would also be the soap company’s (project facilitator’s) responsibility.

## Discussion

Through the formative research, it was clear that awareness and know-how was not limiting handwashing behaviour and that different motivational levers would need to be triggered to encourage people to undertake the behaviour. The team also realised that although promoting the use of bar soap for handwash would seem a possible solution as soap was always present in a home, the general-purpose nature of the product meant that it was always being used for something with a perceived higher priority. There was a clear hierarchy of usage in which use soap for body wash would be the top priority and handwashing was the lowest. This indicated that substantial effort would need to be made to create a product that would not be used for other activities as handwashing would always be de-prioritised.

Consequently, the team developed several possible technologies based on the findings from the Build phase. The development consisted of theoretical concepts and tools being applied to physical prototypes, together with feedback from use by the target demographic. Through this process, a single intervention with multiple form factors for delivery emerged. The design has both a compelling Theory of Change and a reasonable route for manufacture that could be developed in future work.

**Fig 8:**
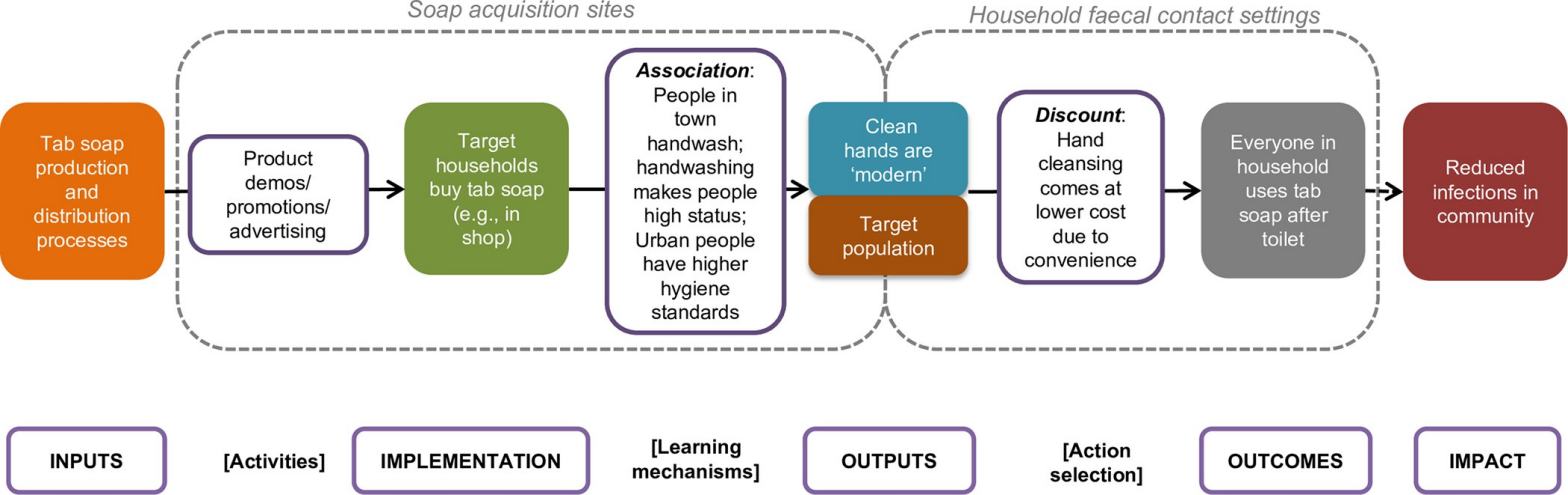
Tab soap theory of change.

The Theory of Change argues that tab soap, made available through business processes of production and distribution, would cause access to this new product, which would in turn cause people in target households to purchase and then use the innovation after the toilet both because it was seen as being part of a ‘modern’ lifestyle, and because it made the practice easier to perform, either in terms of cognitive discounting (don’t have to remember as much) and/or physical discounts (ergonomically more simple) (Fig 8).

The solution is thus thought to be effective because it is a product-service system. That is, the product itself has important features of cleanliness and novelty that influence perceptions and create a reminder but the refill of the soap supply, e.g. through soap sellers in local communities, can create a reinforcing environment for the behaviour and stronger assurance that the product is available. This combination addresses shortcomings in past interventions which have relied on one or the other only, and therefore have not seen desired results.

The largest challenge was that there was no standardisation of context in households, either from the perspective of infrastructure and props or from the standpoint of behavioural routine. The only constant was the diversity in settings. Looking beyond the households at the markets, a similar situation exists. Products are sold at different price points depending on how far they are being sold from the initial distribution point, and the packaging or quantities are continuously modified by sellers according to local factors. These factors made us restrict the brief to in-toilet use, so that some level of standardization of context could be achieved.

Prototyping in this case can be seen as two iterations of a divergent-then-convergent process. First, a conceptual prototype of the intervention was documented with a Theory of Change and a sketch model which addresses broad topics. From this, risks (ways in which the intended Theory of Change could fail) were identified and the conceptual model was updated to mitigate these risks. Next, a divergent process in which specific prototypes for testing each part of the Theory of Change and how these link together was undertaken. These prototypes sought to both embody the final form and mitigate risks associated with practical matters (e.g., build and delivery) as was as theoretical ones (e.g., success of the Theory of Change).

The different kinds of evidence from the field trial—interviews with participants, seller documentation, data logs and field worker interviews—together paint a persuasive picture of use (with an average 1.5 handwashes per household per day). The interviews support the case for these tabs being almost always used for handwashing, which was an important dimension of our objective. The sensor data, although not complete due to technical difficulties, does not appear to be biased by household, and indicates regular use throughout the trial period. As this is the *sine qua non* test of behavioural innovation, we are optimistic about the prospects for use in larger populations with potential for behaviour change.

The Theory of Change was also generally supported and shows some areas for improvement. The product was used in the intended context, successfully compensating for the effort required to remember to wash one’s hands. Environmental factors such as rain and the potential of theft meant that many households kept it in their home (nearly all toilets in this demographic are detached from the home). More might be done to understand how to get the innovation into a household’s toilet. The use of the device within households or in the toilet will also be important to explore in contexts where one owns, rents or shares a toilets, e.g. with other renters or tenants in a compound. The single-use nature of the tabs also meant that there was no cross-contamination and the product was seen as clean. The product was also seen as novel and desirable as evidenced through people showing it off to neighbours.

The simplicity of the design means that it can be manufactured at scale with relatively low costs which appear to be in the price band relevant to the target demographic. However, much of the business model testing needs to be explored further in future work. Specifically, an exploration of modes of delivery, the amount people will be willing to pay to purchase the product and how to motivate this purchase is needed. The product, behaviour and data analysis were aided by innovative use of soap sellers, which can be explored further as the business model continues to develop.

There were several limitations to the study. The creative process was constricted due to a limitation of resources, including time. First, we developed prototypes in London and made minor modifications in the field. A better approach would be to design and test more prototypes in the relevant situation–in Tanzania. In this work, we also prioritised behaviour change and desirability with an eye to technical feasibility and business model viability. An ideal process would allow us to test all aspects further as several questions remain with respect to feasibility and viability. Surely these should be prioritised in subsequent work.

Another limitation was the practical ability to produce test units and measure outcomes. The electronics also worked only for part of the study and offer a useful, if incomplete view of usage. These sensors could be improved to measure use in a better way. There is also an important question about supply and an ability to continue to distribute products in a way that would be compelling to the user to the point that it would generate repeat purchases.

We note that all involved in the study are motivated to see a solution brought to market and serve those in need. Several of the authors remain active in exploring options to develop the concept further, which can include charitable or market-driven solutions to create sustainable supply. These development tracks have differing merits and a goal in much of WASH has long been to motivate people enough to elevate the importance of hygiene that they make the purchase where possible such that any solution does not rely on ongoing charitable contributions which can be subject to disruptions. While this motivation to bring a solution to those who will benefit is right in the eyes of the authors, it also has the potential to generate biased results. To address this, we have made an effort to be transparent and thorough in our communication so the merits of the intervention can be evaluated by others in terms of both output and development process. As is the case with so many interventions, there may be much learning along the way and in the process from which we hope others will benefit.

Finally, the current study did provide compelling early evidence for behavioural change but this iteration of tab soap would not be a solution without a viable and scalable model to bring the soap to many. Indeed, providing free soap which carries novelty to end users is bound to be well received. We did endeavour to create a solution which could be sold at a reasonable price point for the target demographic. However, this does not answer the question of market feasibility. Additional work should explore demand for such a solution particularly around repeat purchasing behaviour and associated use in the home. Within that space an important question is around scarcity and whether the product is abundant enough that it is used regularly as desired or if it is only used periodically.

### Conclusions

The most significant outcome of this study is that a novel hand cleaning product was developed that people in the target demographic are actually willing to use regularly after the toilet. Tab soap appears to have a number of significant advantages over competitor technologies for hand cleansing in this kind of use context:

- Bar and liquid soaps are perceived as cross-contaminating, which makes these solutions disgusting, whereas tab soap is single-use, so one person cannot be put at risk by another’s use of the product.
- Unlike nearly all other forms of soap, it is designed to only be used for washing hands; it is difficult to misuse or use for other purposes.
- Its price-point per use is comparable to alternatives such as a liquid soap bottle push or bar soap rub.
- You can carry a single tab with you like sanitizer, for use anywhere there is water.
- Its use probably requires less water than other forms of soap as the substrate holds water throughout use, lubricating the washing process.
- Unlike soap leaves, ‘singles’ are easier to manipulate and carry, more robust to water and physical damage or degradation, and have post-use value in other contexts.

Families in our trial found a number of innovative ways to store and use the product, pointing to its adaptability as a solution to this difficult behavioural problem. For example, one mother removed a number of tabs at once from her dispenser and gave them to her children to take to school in their backpacks, for use during the day. The containers which were not affixed to a toilet wall were kept in many different locations around the toilet or in the house, suggesting use in more contexts than just post-defecation.

Given that researchers and marketers have long sought to remove the barriers to hand washing after potential contact with faeces, often with little success, this is a welcome achievement. Admittedly, the pilot was small-scale and not of truly long duration, and users were not required to purchase the soap, so further work remains to be done to establish a working business model for this new product. Nevertheless, this is a result that has not previously been observed, and so holds out considerable hope for further success with additional testing. Given the importance of hand hygiene in many public health contexts, including pandemic control, we believe this result should attract widespread interest.

## Supporting information

S1 File Sensor data. Data the 9 households where sensors recorded each time the device was used for at least part of the trial period.
(XLSX)
